# Does a Canadian diabetes curriculum work for future physicians in China? Lessons from the Ottawa Shanghai Joint School of Medicine

**Published:** 2019-03-13

**Authors:** Alexandra Kobza, Ying Dong, Amel Arnaout

**Affiliations:** 1 Faculty of Medicine, University of Ottawa, Ontario, Canada; 2 Faculty of Medicine, Shanghai Jiao Tong University, Shanghai, China

## Abstract

**Background:**

The Ottawa Shanghai Joint School of Medicine (OSJSM) is a campus of the University of Ottawa Medical School in Shanghai, China. This collaboration allowed us to study whether a Canadian curriculum is suitable for the Chinese population. The aim of this study is to evaluate: 1) The OSJSM diabetes curriculum; and 2) The relevancy of the content for the Chinese population.

**Methods:**

The diabetes curriculum content was evaluated using a curriculum comparison between the University of Ottawa, OSJSM, and the Shanghai Jiao Tong School of Medicine (SJTSM). A literature search compared the diabetes populations in Canada and China. Interviews determined how physicians and patients manage diabetes.

**Results:**

The diabetes curriculum at the OSJSM is identical to that of the University of Ottawa. Canada and China have a similar diabetes prevalence, diagnostic criteria, and management. Although both countries utilize the same screening guidelines for diabetes and its complications, patients in Canada are more likely to adhere to these recommendations.

**Conclusion:**

This study suggests that the diabetes content of the University of Ottawa curriculum remains relevant in China. A greater emphasis on the importance of screening for disease complications in the curriculum may facilitate making this a priority for patients and healthcare providers in China.

## Introduction

The present era is one of increasing globalization and the sharing of medical resources is following a similar trajectory. However, it is imperative that those who utilize these resources compare the patient populations and consider their respective differences when applying the results. A similar caution may be appropriate when using medical education teaching materials generated from different countries.

A new partnership between the University of Ottawa Medical School in Canada and the Shanghai Jiao Tong University School of Medicine (SJTUSM) in China represents a unique opportunity to study whether medical education materials can be shared cross-culturally. This partnership was established in 2014 to form the Ottawa-Shanghai Joint School of Medicine (OSJSM), which is a new campus of the University of Ottawa located in Shanghai, China. This program is a commitment to the sharing of medical education and is the first time that a Chinese medical school has adopted a North American training program.^1^

The primary goal of this program is to provide an educational foundation for the development of primary care and family medicine in China. Currently, China’s tertiary care system is centered around specialists, while general practice is not established as an academic discipline in universities.^2^^,^^3^ China is finding this system to be unsustainable for its 1.38 billion population and has recently rolled out a national strategy calling for better access to health care, which includes the development of the primary care sector.^4^^,^^5^ In contrast, 51.9% of physicians in Canada practice family medicine and 85.1% of the Canadian population is linked to a primary care provider.^6^^,7^ Therefore, the SJTUSM believes that implementing a Canadian medical education program aligns with the national strategy to develop a primary care and family medicine model in China.^1^

The notion that higher education institutions are drivers of international change is not new. For decades, universities have been described as “agents of globalization.”^8^^-^^10^ Internationalization of education includes both the sharing of education policy and educational materials. The transference of education policy has been shown to aid in the mobilization of local efforts and financial resources.^11^ On the other hand, the OSJSM project is based on the sharing of educational materials. This transfer of curriculum is bound to come with challenges that Cowen calls “geometries of insertion” which stem from differences in social, cultural, and environmental factors.^12^ The OSJSM has worked to overcome these challenges and the present study further investigates any remaining shortcomings in the transplant of a Canadian medical curriculum to a Chinese medical program.

Students of the OSJSM program are selected from across China upon completion of their high school studies. After a year of introductory science courses, students transition into a four-year medical degree program based on the University of Ottawa’s curriculum. This involves two years of classroom-based teaching, followed by two years of hospital-based training.^1^

Given that the OSJSM is in its infancy, we want to assess whether students are being adequately prepared to serve the Chinese population. As the curriculum of the OSJSM mirrors that of the University of Ottawa Medical School, we evaluated whether the curriculum remains relevant in the context of the Chinese population with respect to prevalent diseases and treatments. Here, we intended to gain insight into this question with a specific emphasis on the diabetes syllabus. The aim of this study was two-fold: 1) To validate the replication of the University of Ottawa Undergraduate Medical Education (UGME) diabetes curriculum at the OSJSM; and 2) To assess how well the content of the OSJSM program will prepare future physicians to meet the needs of the Chinese population they will serve. We approached this from the perspective of the stakeholders who were involved in the design and delivery of the curriculum.

## Methods and participants

### Methods

#### Validating the replication of the Ottawa diabetes curriculum at the OSJSM

To ensure that the classroom-based portion (total of two years) of the diabetes curriculum at the OSJSM was identical to that of Ottawa’s, an analysis was performed of content, hours of teaching dedicated, and qualifications of the instructors. Assessment was completed by a direct comparison of the lecture slides and the course schedule. The local faculty helped in interpretation of the course schedules. All parts of the classroom-based curriculum (lectures, case-based learning, and self-learning modules) were assessed.

#### Suitability of the curriculum to the Chinese population

We assessed suitability in three ways: 1) We performed a PubMed literature search (using keywords: diabetes, Canada, China, statistics) to compare the diabetes population in Canada and China in terms of prevalence of disease and related risk factors, the national strategies in place for diabetes screening, and the availability of treatments and procedures; 2) We compared the diabetes curricula of the University of Ottawa/OSJSM and the traditional medical program at the partnering Chinese University (the SJTUSM) for content, specifically the diabetes classification system, diagnostic criteria, treatments available, disease management guidelines, and the management of complications; this comparison allowed us to assess whether the Canadian curriculum is suitable for the Chinese population under the assumption that the SJTUSM curriculum is the best standard for current management of diabetes in China; and 3) We interviewed students, nurses, physicians and patients individually with the help of a translator to assess stakeholders’ roles and satisfaction in the management of diabetes. We obtained informed consent from all participants. Ethics approval was not warranted for the study, given it is a program evaluation study, as determined by the Ottawa Health Science Network Research Ethics Board.

### Participants

Ten students were randomly selected from the SJTUSM from all years of medical training; all had gone through the diabetes teaching curriculum. Five nurses each with 10+ years of experience and five endocrinologists were selected from the Endocrinology & Metabolism Department at Renji Hospital East Campus. Ten random patients with diabetes were selected from the Endocrinology & Metabolism Inpatient Ward at Renji Hospital East Campus.

## Results

### Validating the replication of the Ottawa diabetes curriculum at the OSJSM

The OSJSM has access to all of the teaching materials of the University of Ottawa. Content experts, chosen from the corresponding SJTUSM faculty, are assigned to each section of the curriculum to review the teaching material and decide whether any additions/deletions/changes need to be made to the curriculum in order to fit the Chinese population. The diabetes curriculum content, comprised of 21 hours of teaching, did not undergo any changes. The only difference found was with the personnel involved in teaching. The OSJSM only has physicians teaching, whereas the University of Ottawa reserves four out of 21 hours of teaching for Registered Dieticians (RD), Registered Nurses (RN), and patients.

### Suitability of the curriculum to the Chinese population

#### Comparing the University of Ottawa/OSJSM curriculum to that of the SJTUSM

The University of Ottawa/OSJSM devotes a greater absolute number of hours to diabetes in the curriculum than SJTUSM (21 vs. 13 hours, respectively), but a smaller fraction of hours relative to the aggregate time spent on internal medicine teaching (3.0% vs. 4.7%, respectively). Within the diabetes curriculum, the two programs assign a similar proportion of time for each sub-topic, as seen in Table 1. The SJTUSM spends comparatively more time on medications (27% vs. 14% of the curriculum), whereas Ottawa/OSJSM spends significantly more time on psychosocial considerations such as impact on quality of life from the patient’s perspective including day to day self-management of the disease and financial costs (10% vs. 0% of the curriculum). Both the Ottawa/OSJSM and SJTUSM curricula discussed the influence of diet on diabetes, albeit not in a culturally specific manner.

**Table 1:** Hours of teaching dedicated to topics of diabetes at the University of Ottawa/OSJSM and at the SJTUSM

Topic	Number of hours (% of total hours spent on diabetes)	
	University of Ottawa/OSJSM	SJTUSM
**Physiology**	1 (5%)	1.33 (10%)
**Pathophysiology: etiology, classification, diagnosis, epidemiology**	2 (10%)	1.66 (13%)
**Medications and Treatment**	3 (14%)	3.5 (27%)
**Complications**	3.5 (17%)	2 (15%)
**Lab Medicine**	3 (14%)	0.5 (4%)
**Case Based Learning**	6.5 (31%)	4 (31%)
**Psychosocial Considerations**	2 (10%)	0 (0%)
**Total**	21 (100%)	13 (100%)

Through student interviews, we found that medical students in the Chinese system acknowledged the burden of diabetes in the country and recognized that a more sedentary lifestyle and the recent increased availability of processed food largely contribute to the increased incidence of disease. Their thoughts on management of diabetes were aligned with those of the Canadian system. In general, students were satisfied with their diabetes curriculum, but identified a need to learn with more case examples.

#### Comparing the Canadian and Chinese diabetes population

Diabetes affects 9.3% of the Canadian population and 11.6% of the Chinese population; whereas the rate of prediabetes in Canada is 22.1% and is estimated at 50.1% in China.^13^^,^^14^ More Canadians than Chinese are overweight (67.7% vs. 35.4%) and obese (35.4% vs. 7.3%), whereas rates of physical inactivity are comparable (25.9% vs. 23.8% respectively).^15^^,^^16^ Table 2 summarizes the state of the diabetes population and care in these two countries.

**Table 2:** Diabetes statistics in Canada and China

	Country	
	Canada	China
**Total Population**	35 940 000	1 376 000 000
**Prevalence of Diabetes & Related Risk Factors**		
਀਀Diabetes	9.3%^14^	11.6%^13^
਀਀Prediabetes	22.1%^9^	50.1%^8^
਀਀Overweight	67.7%	35.4%
਀਀Obesity	30.1%	7.3%
਀਀Physical Inactivity	25.9%	23.8%
**Screening and Diagnosis of Diabetes**	Canadian guidelines^17^	Chinese guidelines^18^
**Screening for complications**	Canadian guidelines^17^	Chinese guidelines^18^
**National Response to Diabetes**		
਀਀Diabetes Registry	No	Yes
਀਀Standard criteria for referral of patients from primary care to higher level of care	Available and fully implemented	Available and partially implemented

Unless otherwise specified diabetes statistics based on diabetes country profiles complied by the World Health Organization in 2016.^15,16^

Diabetes is defined as a fasting plasma glucose (FPG) ≥7 mmol/L or HbA1c≥ 6.5% or 2h plasma glucose (PG) in a 75g oral glucose tolerance test (OGTT) ≥11.1mmol/L or a random PG ≥11.1mmol/L

Prediabetes is defined as FPG 6.1-6.9 mmol/L or HbA1c 6.0-6.4% or 2h PG in a 75g OGTT 7.8-11.0mmol/L

Overweight was defined as the percentage of the population aged 18 or older having a body mass index (BMI)≥25 kg/m^2^

Obesity was defined as the percentage of the population aged 18 or older having a BMI≥30 kg/m^2^

Physical Inactivity was defined as the percentage of the population aged 18 or older who are not performing at least 150 minutes of moderate-intensity physical activity per week.

#### Comparing the management of diabetes in Canada and China

We compared the management of diabetes in the two countries by assessing the curriculum of the University of Ottawa/OSJSM and that of the SJTUSM and through interviews with physicians****. Canadian^17^ and Chinese guidelines^18^ for classification and diagnosis are both based on the World Health Organization.^19^ Canadian^17^ and Chinese^18^ screening guidelines for the disease and its complications are both based on the International Diabetes Federation recommendations.^20^ Screening is the responsibility of family doctors in Canada, whereas in China, there are no physicians who are directly responsible for ensuring patients adhere to screening guidelines and therefore patients have to seek out these tests independently. Although there are no numbers to support this, physicians in China hypothesize that much less of the population adheres to the screening guidelines recommended by physicians.

China currently does not have the infrastructure to administer screening and follow-up in a widespread manner. By October 2010, health records had only been established for 36% of the urban population and 24% of the rural population.^2^ No estimates could be found for the number of patients who visit primary care centres for frequent well-checks. Through physician interviews we learned that only a small percentage of patients choose to have a well-check every 1-2 years, often to meet workplace requirements. Patients with diabetes are diagnosed when they present to the hospital with symptoms, incidentally, or infrequently through well-checks.

Based on curriculum content and physician interviews, both Canada and China consider the same diabetes complications. These are: acute complications (diabetic ketoacidosis, hyperosmotic hyperglycemic state, and hypoglycemia), chronic macrovascular complications (cerebral vascular disease, coronary heart disease, and peripheral vascular disease) and chronic microvascular disease (diabetic retinopathy, renal disease, and neuropathy). In Canada, the managing physician (either family doctor or endocrinologist) arranges the screening tests, whereas in China, patients organize this independently. The rates of complications in Canada are documented and well-studied,^16^ while we could not find data on the rates in China.

#### Treatments used

All medications and procedures offered by physicians in Canada and China were identical, as seen in Table 3, with the exception of one traditional Chinese medication called Tangmaikang, a herbal formula which is seldom prescribed, reported to have antihyperglycemic effect mediated by increasing insulin sensitivity.^15^^,^^16^ Of note, this medication was not included in either the OSJSM or the SJTUSM curriculum; however, in China, it was mentioned by Endocrinologists in a clinical setting.

**Table 3:** Availability of medications and procedures in the Public Health Sector in Canada and China

	Country	
Medications*	Canada	China
Insulin		
਀਀Intermediate acting	Available	Available
਀਀Peakless Basal	Commonly used	Commonly used
਀਀Short Acting	Available	Available
਀਀Rapid Acting	Commonly used	Commonly used
਀਀Premixed insulin	Available	Commonly used
Oral Medication		
਀਀Acarbose	Rarely used	Commonly used
਀਀Metformin	Commonly used	Commonly used
਀਀Thiazolidinediones	Rarely used	Rarely used
਀਀Sulfonylureas	Available	Commonly used
਀਀Meglitinides	Available	Commonly used
਀਀GLP-1 agonists	Available	Available
਀਀DPP-IV Inhibitors	Commonly used	Available
਀਀SGLT-2 Inhibitors	Commonly used	Rarely used
Traditional Chinese Medicine		
਀਀Tangmaikang	Not used	Rarely used
Lifestyle Modifications		
਀਀Diet	Commonly used	Commonly used
਀਀Exercise	Commonly used	Available
**Procedures**		
Retinal photocoagulation	Yes	Yes
Renal replacement therapy by dialysis	Yes	Yes
Renal replacement therapy by transplantation	Yes	Yes

* Specifications for frequency of treatment use in Canada is based on judgement of Endocrinologists at The Ottawa Hospital for Canada and Endocrinologists at Renji Hospital in Shanghai for China

Availability of procedures based on diabetes country profiles complied by the World Health Organization in 2016.^15,16^

#### Role of health care professionals in the management of diabetes patients

A major distinction between the Chinese and the Canadian health care systems lies in how patients access the services of health care professionals. Patients in Canada enter the health care system through a family or emergency physician and all further contact with health care professionals is through a referral system. In contrast, all members of the health care team in China are accessed autonomously by the patient, often in the absence of guidance and/or communication between members. Furthermore, there are minor differences in the roles of health care professionals who are involved in the management of diabetes. We evaluated the roles of endocrinologists, family doctors, nurses, dieticians, social workers, and foot care specialists. Diabetes nurses provide one-on-one education to patients in Canada, whereas at Renji Hospital in Shanghai they run weekly patient classes on diet, exercise, psychology, insulin injections and blood sugar monitoring. Dieticians have a professional degree in Canada, while in China they are a medical specialty and have their own outpatient clinics. Social work is not a service in China, nor is the foot care service. In China, family members do most foot care, and surgery cares for severe cases.

## Discussion

The Ottawa-Shanghai Joint School of Medicine (OSJSM) is a branch of the University of Ottawa Medical School located in Shanghai, China. It is a product of a partnership between the University of Ottawa Medical School and the Shanghai Jiao Tong University School of Medicine, which began in 2014. Given that the curriculum of the OSJSM remained unchanged from the University of Ottawa UGME curriculum, we assessed whether this Canadian program remains relevant in the context of the Chinese population, specifically for diabetes. We evaluated the similarity of diabetes education in Canada and China through a curriculum comparison with the SJTUSM, a literature review of diabetes in the two countries, and interviews with medical students, endocrinologists, diabetes nurses, and patients.

The SJTUSM curriculum was very similar to that of Ottawa/OSJSM. The Ottawa/OSJSM dedicates more hours to diabetes than the SJTUSM, but a smaller percentage of time relative to all topics in internal medicine. The SJTUSM spends proportionally more time on medications and treatment, while the Ottawa/OSJSM program spends more time on psychosocial concerns.

There is a similar prevalence of diabetes in Canada and China, while the estimated rates of prediabetes in China (50.1%) are more than two-fold those in Canada (22.1%).^13^^,^^14^ The correlation between being overweight/obese and having diabetes is observed in both countries, although the percentage of those who have higher BMI is lower in China while the rates of physical inactivity are similar in both countries.^15^^,16^ This strongly suggests that genetic heterogeneity and other lifestyle factors such as dietary considerations are responsible for the significantly higher rates of prediabetes in China. As prediabetes is the main risk factor for future development of diabetes, China is on the verge of an epidemic unless successful screening and prevention strategies are implemented. These are factors that are important for future Chinese physicians to be aware of and therefore should be emphasized in the OSJSM curriculum.

Diabetes classification, diagnosis, and screening guidelines (for both diabetes and its complications) are established by Diabetes Canada^17^ and the Chinese Diabetes Association,^18^ both of which are in alignment with the World Health Organization classification and diagnostic criteria and the International Diabetes Federation screening and management recommendations.^19^^,^^20^ Both countries screen patients for the same complications, including acute, macrovascular chronic, and microvascular chronic complications. However, unlike in China, screening for the disease and its complications is a priority in Canadian diabetes management, where all tests are ordered by the managing physician and results at a population level are widely studied.^21^ In contrast, physician and patient interviews suggested that in China screening guidelines for the disease and its complications are not followed as closely. Patients are informed of optimal screening tests for vascular complications by their endocrinologists; however, not all patients are compliant with these recommendations; seemingly due to a lack of awareness of the gravity of the condition.

Patient interviews revealed that the majority of patients only undergo screening for complications when admitted to the inpatient endocrinology ward. Furthermore, diabetes complication rates in the Chinese population could not be found. In light of this, placing an additional emphasis on the importance of complication screening in the OSJSM curriculum may encourage health care professionals to make this a priority. It may also promote the development of health care infrastructure to allow for organized follow-up in the context of the planned primary care model.

Structured follow-up would facilitate more communication between health care professionals. A similar multidisciplinary team for diabetes management exists in both Canada and China, including endocrinologists, family doctors, dieticians and nurses (but excluding social work and foot care in the latter). Although patients may receive guidance from their endocrinologists as to which service they should access, it was evident that in general, patients make use of fewer interprofessional services in China than in Canada.

The medications and procedures available for the treatment of diabetes are almost identical in both countries. The only addition was one traditional Chinese medication, tangmaikang, which is rarely prescribed by endocrinologists in China and only in the context of mild diabetes. All things considered, the University of Ottawa/OSJSM’s approach to the treatment of diabetes remains relevant for the Chinese population.

Our study has several strengths, the most important of which is its contribution to the advancement of international partnerships. It is a proof-of-concept that it is possible to transplant a medical curriculum from one country to another with a different demographic and culture. It remains to be seen whether this initiative manifests into the development of the primary health care sector in China. Another strength of this study is that it highlights the areas of need in the Chinese health care system at the level of both diabetes education and clinical practice. With respect to diabetes education, the Chinese SJTUSM and Canadian-based OSJSM curricula both fail to address factors specific to the Chinese population such as the prevalence of diabetes/prediabetes and genetic and dietary/lifestyle considerations. With respect to clinical practice, the aforementioned shortcomings in screening and prevention stem directly from the Chinese health care system’s current lack of primary care infrastructure further substantiating the salience of this partnership.

The main limitation of the study lies in the fact that the suitability of the Canadian diabetes curriculum was only assessed in the context of one Chinese university medical curriculum and one hospital in China. There are likely differences between medical education as well as diabetes management practices between different cities in China and more so between urban and rural centres. Therefore, it is unclear whether our results are applicable throughout China.

Future research could also evaluate whether other topics in the University of Ottawa medical curriculum adequately align with the Chinese population in terms of demographics, risk factors, lifestyle considerations, disease prevention and management. Furthermore, it would be of interest to compare the perceptions of the medical students who participate in the adopted Canadian undergraduate medical curriculum to those in the traditional program, in particular whether the program manifests into greater interest in primary care medicine should a postgraduate program in family medicine develop in their country. If such a project succeeds it would demonstrate how changes to health care systems can begin with shifts in education.

### Conclusion

This study suggests that it is appropriate and advantageous to educate future physicians in China using the diabetes syllabus of the University of Ottawa, given China’s national strategy to develop the primary care sector. A primary care model that draws on principles of the Canadian system may help guide China in assuming the large burden of prediabetes screening and implementation of preventative therapies. Regular follow up of patients with established disease is also essential to effectively prevent complications. A greater emphasis on the importance of diabetes screening in the OSJSM curriculum may translate to earlier recognition of the disease and its complications, thereby improving patient outcomes.
